# A method based on ^10^B isotope tracer for seepage detection in salt lakes

**DOI:** 10.1039/d5ra02724a

**Published:** 2025-08-26

**Authors:** Jikui Hu, Yufei Deng, Lingfen Wang, Huang Wang, Liangliang Zhao, Liling Lu, Zhongjian Liu, Qiang Wang, Minshan Shi

**Affiliations:** a Key Laboratory of Coal Cleaning Conversion and Chemical Engineering Process, Xinjiang Uyghur Autonomous Region, College of Chemical Engineering, Xinjiang University Urumqi Xinjiang 830017 China wangqiang@xju.edu.cn 458804348@qq.com; b Geology Institute of China Chemical Geology and Mine Bureau Beijing 100101 China; c SDIC Xinjiang Luobupo Potash CO., Ltd Hami 839000 China

## Abstract

To address the technical challenges of monitoring brine seepage in salt lakes, this study pioneers the application of ^10^B isotope tracer to seepage detection, establishing a high-precision monitoring system that provides scientific foundations for precise seepage channel identification and flow field characterization. Systematic laboratory experiments validate the exceptional performance of the ^10^B tracer, including ultra-trace detection sensitivity of 10^−9^, a stable recovery rate of 92.8–106.5%, adsorption loss below 6.31%, and light transmittance and acid-base resistance retention rates exceeding 95%, confirming its reliability and applicability in complex high-salinity brine systems. Compared to the fluorescein control test, the ^10^B tracer demonstrated greater advantages in adsorption rate and buffered pH resistance. Mobility experiments further reveal inert-like migration characteristics of the ^10^B tracer across media, with a breakthrough curve highly similar that of chloride ions. Its rapid transport capability and low retention loss below 20% enable real-time tracking of seepage pathways, offering critical technical support for dynamic flow field analysis. Field experiments reveals that the main seepage path in the salt field leakage system is 2 → 1 → 6, dominated by a fracture system with rapid flow and high connectivity. The secondary seepage path is 2 → 1 → 4 → 6, formed by the interaction of fractures and micro-pores, characterized by fluctuating concentrations and low transmission efficiency. The rapid response and stable flow of the main path highlight the hydraulic dominance of the fracture system, while the oscillating behavior of the secondary path reflects geological heterogeneity and uneven pore distribution. For well 3, Peclet number calculation yielded Pe >1, with absent ^10^B tracer inflow primarily caused by an auxiliary plugging mechanism. Delayed responses and weak signals in edge wells including well 5 and well 7 further confirm permeability barriers. These findings provide critical guidance for precise seepage control engineering.

## Abstract 2

This study pioneers the use of ^10^B isotope tracer for brine seepage monitoring in salt lakes, establishing a high-precision system to identify seepage channels and characterize flow fields.

## Graphical abstract

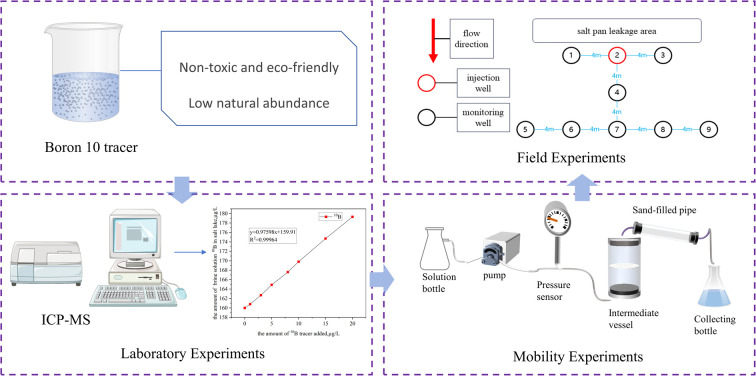

## 1. Introduction

Modern salt lakes in China are rich in inorganic salt resources within their brines, containing not only potassium, sodium, chlorine, and magnesium but also substantial reserves of lithium, boron, rubidium, cesium, iodine, and bromine.^1,2^ However, during potassium salt extraction through well mining, brine transportation *via* channels and solar evaporation in salt pans frequently induce groundwater leakage,^3^ resulting in partial brine loss into subsurface aquifers before the brine solidifying into mineral deposits. Currently, the lack of a systematic groundwater monitoring system in the salt pan area obscures the migration pathways and dispersion extent of leaked brine, which urgently requiring scientifically robust technical approaches to characterize subsurface flow fields, quantify brine transport directions and leakage flux rates, and provide theoretical foundations for resource recovery.

The identification of salt pan leakage poses a common technical challenge in current brine resource development, primarily manifested in three aspects. First, existing leakage monitoring techniques^4,5^ such as groundwater monitoring, electrical resistivity tomography, and elastic wave detection generally suffer from insufficient detection accuracy and high operational complexity. Second, the heterogeneous permeability of natural clay impermeable layers in salt pan areas, particularly the lack of engineered anti-seepage measures^6^ in primary evaporation units such as sodium salt ponds, exacerbates brine leakage risks. Third, dynamic hydrogeological alterations^7^ induced by large-scale brine extraction operations lead to spatiotemporal variability in parameters such as water abundance and permeability within mining zones, significantly complicating the precise identification of leakage pathways.

Isotope tracing technology, renowned for its strong capability in tracking material migration and high sensitivity, has been widely applied in seepage detection for hydraulic engineering projects. International studies demonstrate that Acworth *et al.*^8^ successfully measured groundwater flow rates in coastal aquifers of New South Wales, Australia, using ^82^Br tracer; the Grabowski team^9^ employed ^234^U/^238^U ratios to delineate water cycling processes in central Poland; Birkle *et al.*^10^ investigated deep groundwater movement directions in Mexico's Samaria-Sitio Grande oilfield *via*^18^O/^2^H analysis; and Johnson *et al.*^11^ utilized ^87^Sr/^86^Sr ratios to study groundwater runoff in the Snake River Plain aquifer system. Zhu *et al.*^12^ tracked the magmatic process with Zr isotopes, demonstrating the primary controlling role of fluids in the fractionation of Zr isotopes during the crystal-magmatic-fluid differentiation process. Gong *et al.*^13^ used mercury stable isotopes to track volcanic activities in geological records. Tian *et al.*^14^ used potassium isotopes to trace the genesis of island arc rocks. However, a specific tracer system tailored for high-salinity brine leakage remains absent. Following the SY/T 5925-2012 standard “Guidelines for Chemical Tracer Selection in Oilfield Water Injection”,^15^ systematically screened 40 common elements and their isotopes, comprehensively evaluating core parameters including radioactivity, isotopic abundance, toxicity levels, and aqueous solubility characteristics, followed by a phased multicriteria filtration process.^16–18^ Firstly, elements with high radiation risks or acute toxicity were excluded based on radioactivity thresholds and biological toxicity limits. Secondly, targets exhibiting stable dissolution and compatibility with analytical techniques, such as spectroscopy and mass spectrometry were selected using aqueous solubility data. Subsequently, candidates with significant environmental background interference or exceeding instrumental detection limits were eliminated by comparing ambient background concentrations with detection thresholds. Finally, economic viability was optimized by assessing industrial production scale, raw material costs, and supply chain stability. Through this progressive screening protocol, the ^10^B isotopic tracer emerged as the optimal candidate due to its negligible natural radioactivity, controllable biotoxicity, moderate aqueous solubility, low environmental background interference, and well-established isotope separation technology coupled with a robust commercial supply chain. Furthermore, the ^10^B isotopic tracer demonstrates distinct advantages over nanoparticle and DNA tracers in both sensitivity and cost-effectiveness. Specifically, its primary benefit lies in ultrahigh detection sensitivity: neutron activation analysis enables precise quantification of trace ^10^B at parts-per-billion levels, significantly surpassing the optical detection limits of nanoparticles and the amplification dependency of DNA techniques. Economically, despite natural scarcity, mature enrichment processes ensure ^10^B is more cost-efficient at scale than complex synthetic nanoparticles or high-purity DNA tags. Critically, ^10^B exhibits chemical inertness, resisting thermal, pH, and biodegradation effects, whereas nanoparticles are susceptible to aggregation and DNA tracers are prone to enzymatic degradation. For long-term monitoring applications, the inherent physicochemical stability of ^10^B ensures data integrity and eliminates risks associated with nanoparticle ecotoxicity or DNA mutation. Therefore, ^10^B represents a technically robust and economically sustainable solution for demanding applications such as deep-earth exploration and hydrology. Through integrated laboratory and field experiments, we evaluate its leakage identification efficacy and establish a tracer methodology specifically designed for salt lake brine seepage detection, providing a reliable tool for investigating spatial heterogeneity in subsurface flow fields.

## 2. Experiment and method

### 2.1 Reagents and instruments

Inductively Coupled Plasma Mass Spectrometer, Agilent 7900 ICP-MS, Agilent Technologies; UV-visible Spectrophotometer, Model UV-3600, Shimadzu Corporation; Fluorescence spectrometer, model FLSP920, Edinburgh Company; ^10^B Isotopic Tracer, Purity: ≥99.9%, Wuhan Eastop Sci-Tech; ^10^B Isotope Reference Material, GBW 08607, National Institute of Metrology China; Polyethersulfone Membrane Filter, Pore Size 0.22 μm, Tianjin Leading Laboratory Equipment; Sodium Chloride, Fluorescein and Nitric Acid, Analytical Reagent Grade, Sinopharm Chemical Reagent; Brine and silt samples, collected from Lop Nur Salt Lake.

### 2.2 Laboratory detection experiments

This study first conducted vacuum filtration of salt lake brine using 0.22 μm membrane filters. The background concentration of the ^10^B tracer in the brine was determined through inductively coupled plasma mass spectrometry combined with a dynamic correction model. Following the U.S. EPA standard method,^19^ the method detection limit and quantitation limit were established as 3*σ* and 10*σ*, respectively, *via* seven parallel blank experiments. A standard addition method was then applied by spiking the brine system with ^10^B standard solutions at gradient concentrations ranging from 0 to 20.0 μg L^−1^ in 5.0 μg L^−1^ increments, followed by measurement of the ^10^B/^11^B isotopic ratio.

Following the preparation of ^10^B tracer solutions with concentration gradients ranging from 0 to 20 μg L^−1^ in 5 μg L^−1^ increments, these solutions were mixed with brine at a 1 : 1 volume ratio. After 24 hours of dark incubation at 25 °C, the transmittance within the visible light spectrum was measured using an ultraviolet-visible spectrophotometer. Systems with adjusted pH levels were analyzed to determine the variation rate of the ^10^B concentration and the ^10^B/^11^B isotopic ratio, thereby assessing the photochemical stability of the tracer. Further validation involved injecting ^10^B isotopic tracer, at five concentration gradients ranging from 0 to 20 μg L^−1^, into both natural salt lake brine and artificially prepared Ca^2+^ and Mg^2+^ brine with comparable concentrations. Recovery rates were calculated based on the linear relationship between the inductively coupled plasma mass spectrometry response values and the calibration curve, which has confirmed the accuracy of the tracer quantification.

Finally, for fine salt particles smaller than 1 mm, coarse salt particles of 1–3 mm, and powdery samples, add a tracer solution with a concentration gradient of 0–20 μg L^−1^ at a mass-to-volume ratio of 1 : 3. The mixture was oscillated at a constant temperature of 25 °C in a centrifuge at 300 rpm for 48 hours, followed by centrifugation at 4000 rpm for 15 minutes to separate the liquid phase. After filtration through a membrane filter, the residual ^10^B concentration was measured to calculate the adsorption rate of the tracer by the medium.

Fluorescein tracer was added as a parallel control in this study and detected using a fluorescence spectrometer. Changes in fluorescence intensity were monitored to obtain performance metrics for fluorescein, including linear range, pH resistance in buffer, transmittance, static retention rate, recovery rate, and adsorption rate. These metrics were then comparatively analyzed against those of the ^10^B tracer using radar plots.

### 2.3 Mobility experiments

**Fig. 1:**
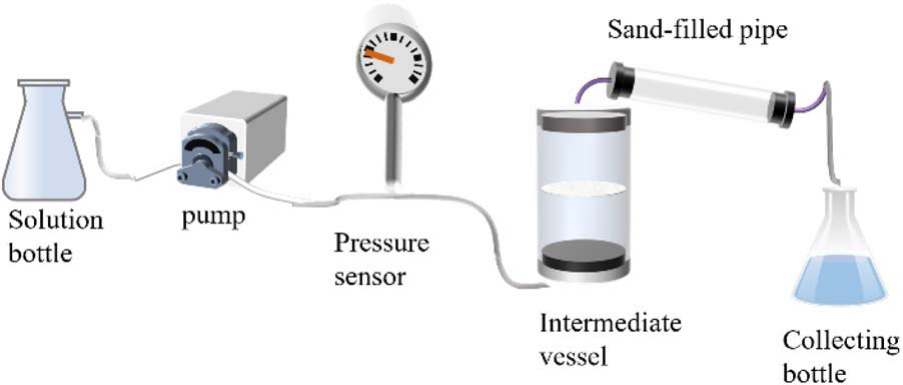
Schematic diagram of the mobility experimental setup.

This study conducted displacement experiments using three solutions: a 100 mg per L NaCl solution as the displacing fluid, a NaCl solution with a chloride ion concentration of 1000 mg L^−1^ as the inert reference tracer, and a candidate ^10^B tracer solution with an initial concentration of 20 mg L^−1^ calibrated *via* inductively coupled plasma mass spectrometry. A sand-packed tube model with pore volumes (pv) detailed in Table 1 was employed. Formation-simulated water injection was performed at 4 m^3^ h^−1^, sequentially injecting 2 pore volumes of tracer solution followed by displacing fluid. Effluent samples were collected at 0.1 pore volume intervals, and ^10^B and Cl^−^ concentrations were determined. Breakthrough-elution comparative curves for the inert Cl^−^ baseline and ^10^B tracer were plotted with injected pore volume as the *x*-axis and normalized concentration *C*/*C*_0_ as the *y*-axis. The depletion rate of the ^10^B isotopic solution was calculated by analyzing effluent-to-initial tracer concentrations. The experimental system, illustrated in Fig. 1, consisted of a high-pressure constant-rate pump, pressure transducers, intermediate containers, and the sand-packed tube. Real-time pressure monitoring ensured stable displacement processes throughout the experiment.

**Table 1:** Pore volume characteristics of different packing materials in sand-packed columns

Name	Tube weight (g)	Weight after sand filling (g)	Weight after brine injection (g)	Pore volume (mL)
Fine salt	2214.2	2354.2	2403.0	48.8
Kosher salt	2214.3	2368.7	2411.8	43.1
Silt	2214.5	2430.9	2458.1	37.2

### 2.4 Field experiments

**Fig. 2:**
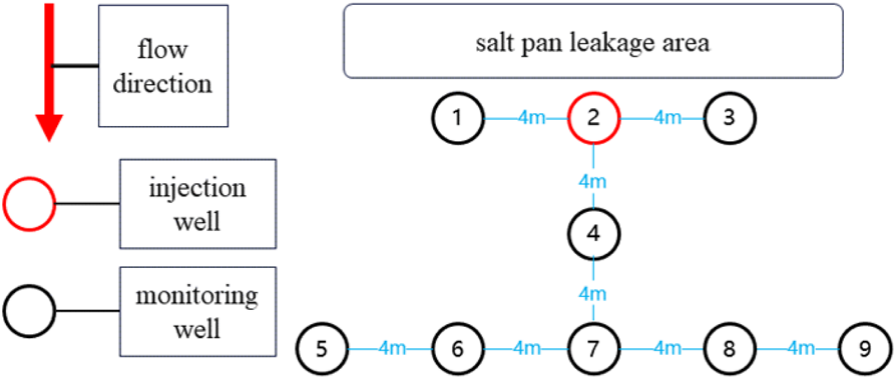
Interwell location map for field-scale tracer applicability test.

This study deployed nine monitoring wells with a depth of 1.2 m in the salt lake test area, with their spatial distribution shown in Fig. 2 the geological parameters, taking well 3 as an example, were obtained through field measurements and pumping tests, and calculated using formula (3): drawdown *s* = 1.2 m, pumping rate *Q* = 2.592 m^3^ d^−1^, time *t* = 6.94 × 10^−4^ d, and radial distance *r* = 4 m. Iterative calculation using the Theis formula yielded a hydraulic conductivity *T* = 0.313 m^2^ d^−1^ and a storativity *S* = 5.44 × 10^−6^. Given the aquifer thickness *M* = 5 m and the relationship *T* = *KM*, the permeability coefficient *K* is calculated as 0.06 m d^−1^. This *K* value is typical for clayey soils. Well 2 was designated as the injection well for the ^10^B isotopic tracer, while the remaining eight served as monitoring wells. Based on hydrogeological survey data, the regional groundwater flow direction was preliminarily determined as north to south. After injecting 50 g of ^10^B isotopic tracer solution into well 2, a standardized sampling protocol was implemented; brine samples at 1.2 m depth were collected twice daily from monitoring well ports, with pre-sampling purging to remove residual brine and ensure acquisition of fresh seepage fluid. Collected samples were filtered through 0.22 μm membrane filters, aliquoted into vials, stored under dark and low-temperature conditions, and accompanied by timestamped records. Continuous monitoring commenced 24 hours post-injection and lasted for 20 days, yielding 320 valid samples. All samples underwent ^10^B/^11^B isotopic ratio analysis *via* inductively coupled plasma mass spectrometry within 20 days of collection to ensure timeliness in seepage pathway tracking.

### 2.5 Data analysis

The two stable isotopes of boron, ^10^B and ^11^B, exhibit significant isotopic fractionation effects during migration due to their mass differences. Leveraging the high-precision isotopic detection capability of inductively coupled plasma mass spectrometry and calibrated against the isotopic abundance reference values of standard material^20^ GBW 08607, a dynamic correction model for isotopic ratios, established *via* formula (1), allows precise quantitative analysis of ^10^B concentration and the ^10^B/^11^B isotopic ratio during experiments.^21,22^ Real-time instrumental drift calibration maintains single-sample analysis uncertainty below 0.2‰, complemented by an online cleaning procedure with 0.1 mol per L HNO_3_(aq) to suppress memory effects to background levels, thereby ensuring the reliability of computational results.1
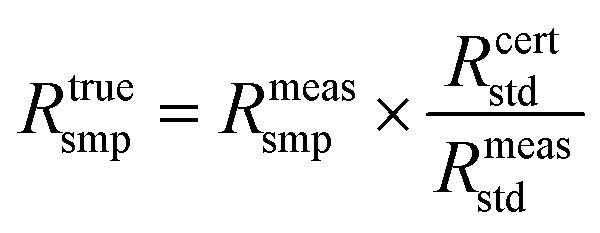


Here, *R*^true^_smp_ denotes the corrected isotope ratio of the sample, *R*^meas^_smp_ refers to the directly measured isotope ratio of the sample, *R*^cert^_std_ represents the certified theoretical isotope ratio of the standard material, and *R*^meas^_std_ indicates the measured isotope ratio of the adjacent standard material.

In addition, this study calculated the Peclet number, as shown in formula (2), which is a dimensionless number characterizing the relative importance of convective and diffusive transport in a fluid.2
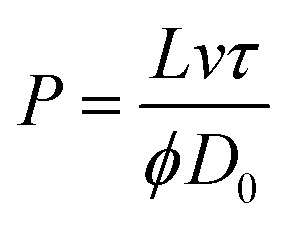


Here, *L* denotes the feature length, *m*; *v* denotes the Darcy velocity, m s^−1^; *τ* denotes the degree of curvature, dimensionless; *ϕ* denotes the porosity, dimensionless; *D*_0_ denotes the diffusion coefficient of the free liquid phase, m^2^ s^−1^.

To better understand geological parameters, we introduced the Tess formula, as shown in formula (3).3
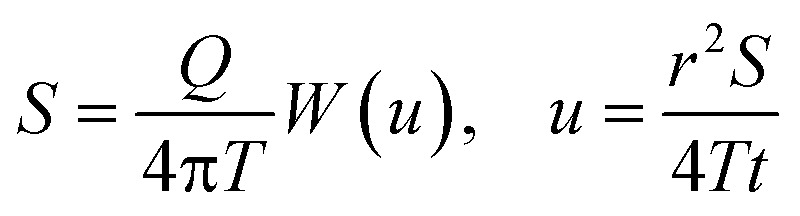
Here, *S* denotes drawdown, m; *Q* is the pumping rate, m^3^ day^−1^; *T* represents hydraulic conductivity, m^2^ day^−1^; *W*(*u*) is the well function, dimensionless; *r* indicates radial distance to the observation well, m; *S* refers to storativity, dimensionless; and *t* stands for pumping duration, *s*.

## 3. Results and discussion

### 3.1 Performance validation and applicability analysis of laboratory detection

**Fig. 3:**
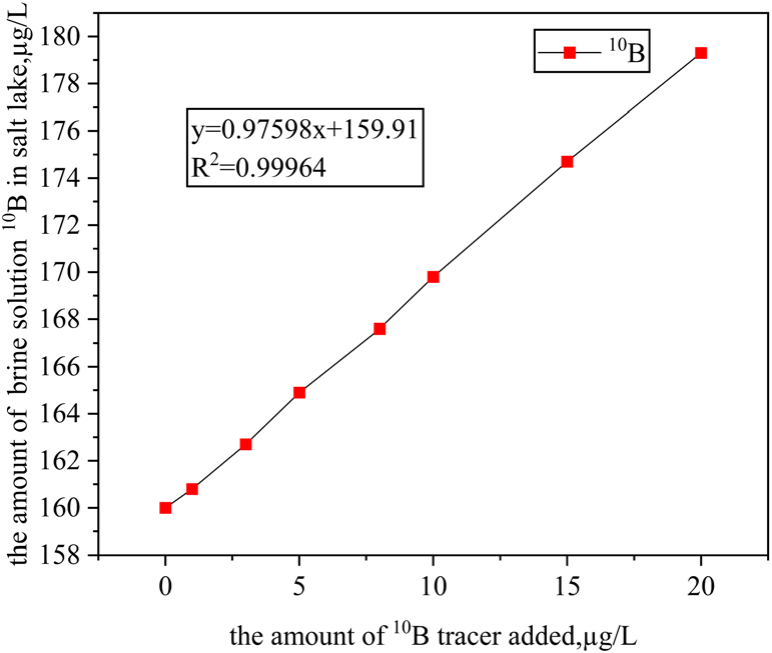
Standard addition curve.

This study validated the detection performance of the ^10^B isotopic tracer. Results demonstrated a high linear correlation between the ^10^B/^11^B isotopic ratio and inductively coupled plasma mass spectrometry signal intensity, with a linear correlation coefficient *R*^2^ exceeding 0.999. The method exhibited a detection limit of 1.2 μg L^−1^ and a quantitation limit of 4 μg L^−1^, calculated based on 3*σ* and 10*σ* standard deviations, confirming ultra-trace detection capability at the 10^−9^ level. In laboratory applicability tests using a salt lake brine system with a background ^10^B concentration of 160 μg L^−1^, the addition of 5 μg L^−1^ of ^10^B tracer induced significant variations in signal intensity, fully meeting the sensitivity requirements for leak monitoring.^23^ These results indicate that the ^10^B isotopic tracer effectively distinguishes background fluctuations from artificially introduced trace level changes, providing critical experimental evidence for optimising tracer dosage in practical engineering applications. Detailed experimental results are shown in Fig. 3.

**Fig. 4:**
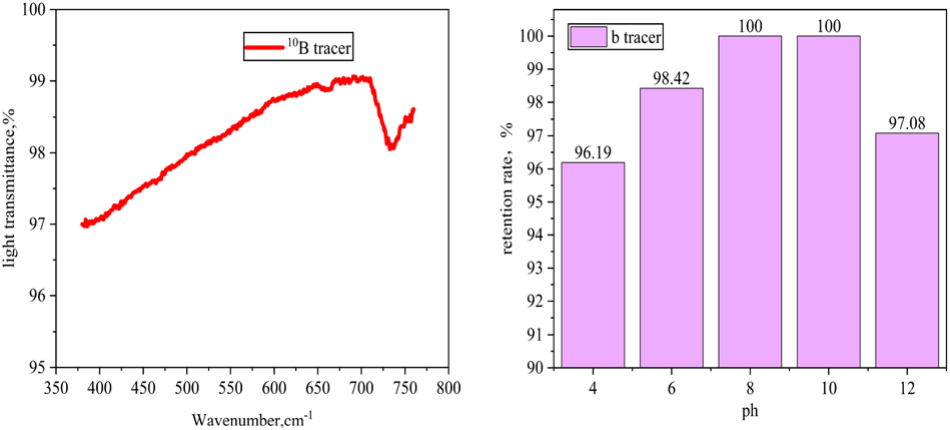
(a) Transmittance plot; (b) Retention plot for different pH values.

After being mixed with salt lake brine for 24 hours, the ^10^B tracer demonstrated excellent optical stability, with a transmittance maintained at 98.2% ± 0.3%. The isotopic ratio deviation remained below 2%, indicating no turbidity or suspended particles in the solution, which reflects good stability. Additionally, under dynamic pH conditions ranging from 4 to 12, the retention rate of the ^10^B tracer remained stable at over 96%, highlighting its excellent acid and alkali resistance, as shown in Fig. 4.

**Fig. 5:**
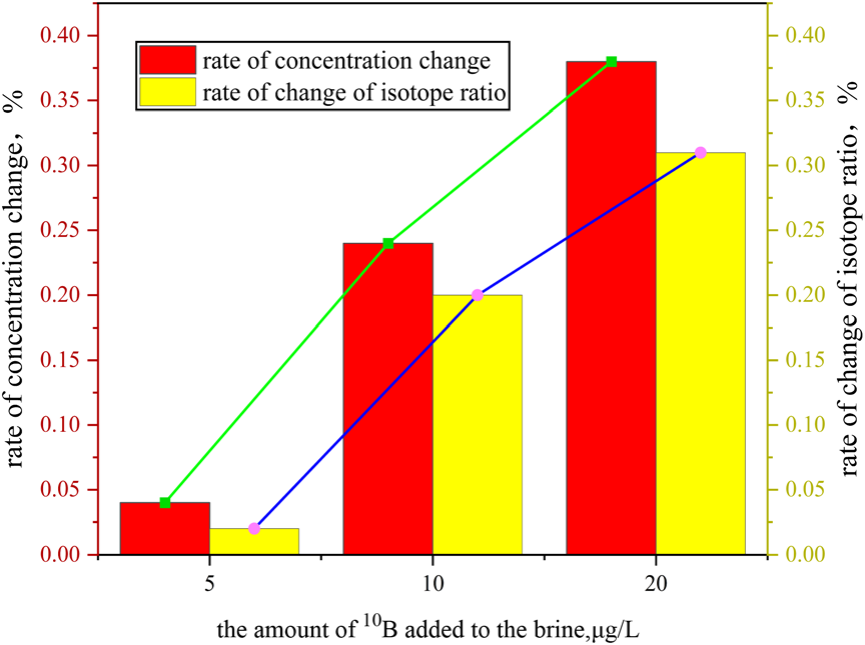
Retention rate stability experiment.

As shown in Fig. 5, when the concentration of the ^10^B tracer was gradually increased from 5 μg L^−1^ to 20 μg L^−1^, the changes in the ^10^B tracer concentration and isotopic ratio remained below 0.38%, demonstrating its excellent stability. As the concentration increased, the isotopic ratio deviation rose from 0.02% to 0.31%, indicating that the tracer system at lower concentrations exhibits superior phase stability.^24–26^ Under low-concentration conditions, boric acid molecules are uniformly dispersed in the brine system in the form of monomeric B(OH)_3_/B(OH)_4_^−^, with weak intermolecular interactions, bringing the system closer to an ideal solution state and enhancing phase stability. When the concentration is relatively high, boric acid molecules self-assemble into polymeric associates of the [Bn(OH)_3_*n*]^*n*−^ type through hydrogen bonding between hydroxyl groups. The accumulation of these polymers may lead to localized supersaturation in the solution, potentially causing spontaneous crystallization or precipitation. Therefore, the experimental results provide a basis for optimizing the tracer dosage strategy.

**Fig. 6:**
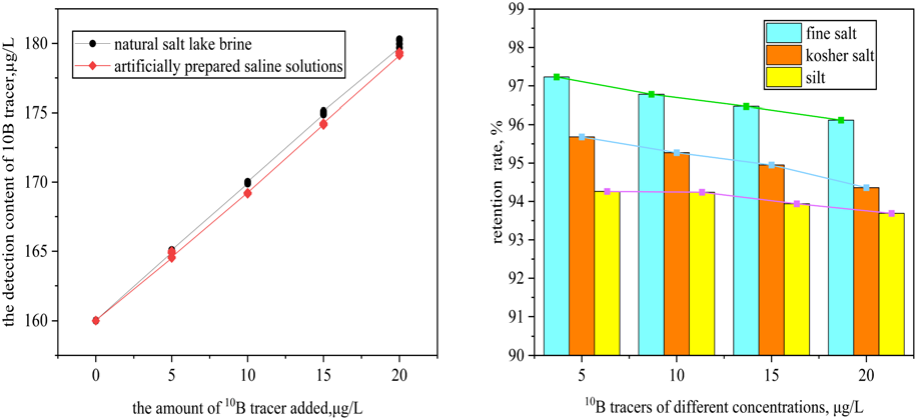
(a) Spiked recovery; (b) adsorption loss rate of different media.

As shown in Fig. 6(a), the ^10^B tracer exhibited high recovery rates ranging from 92.8% to 106.5% across the tested concentration range of 0 to 20 μg L^−1^. As a key indicator for evaluating the accuracy of a method in chemical analysis, the recovery rate directly reflects the degree of closeness between the measured value and the true value. The recovery rate obtained in this study complies with the permissible range of 90–110% set by the US Geological Survey for trace element analysis standards,^27^ systematically verifying the reliability of the experimental system. This result confirms the accuracy of ICP-MS technology in quantitative analysis from a methodological perspective and indicates that the established method has ideal detection efficiency.

As shown in Fig. 6(b), the adsorption rate of the ^10^B isotope tracer increases progressively with higher concentration. Furthermore, at a concentration of 20 μg L^−1^, the inhibition loss rates are remarkably low, measuring 3.88% ± 0.18% in fine salt, 5.74% ± 0.31% in coarse salt, and 6.31% ± 0.42% in sludge media. These exceptionally low values demonstrate its exceptional anti-adsorption properties. The study further revealed that this anti-adsorption behaviour arises from synergistic multiscale physicochemical mechanisms. In fine salt media, the surface negative charges of particles and the electrostatic repulsion between boric acid anions [B(OH)_4_^−^] significantly reduce the probability of physical adsorption.^28^ While kosher salt particles possess porous structures capable of temporarily trapping certain substances, their lack of effective binding sites hinders the formation of stable chemical bonds.^29^ Although clay minerals in silt exhibit strong adsorption capacities, the lighter ^10^B isotope tends to detach more readily from mineral surfaces due to its structural characteristics.^30^ Consequently, ^10^B in salt lakes demonstrates enhanced permeability through media and maintains superior migration capacity.

### 3.2 Analysis of transport dynamics and retardation mechanisms in mobility experiments

**Fig. 7:**
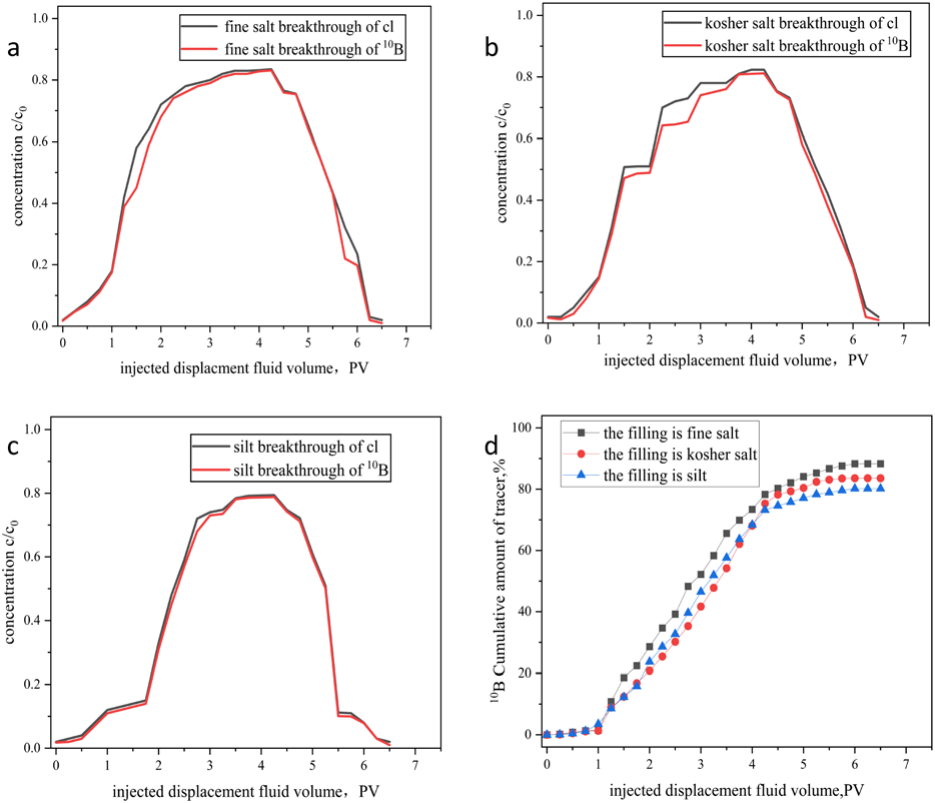
Breakthrough curves of ^10^B tracer and Cl^−^ in porous media: (a) fine salt; (b) kosher salt; (c) silt; (d) effluent concentration *versus* injected pore volume across packing materials.

By comparing the breakthrough curves of the ^10^B tracer and Cl^−^ in Fig. 7(a)–(c), we found that the two curves were highly similar. This shows that the ^10^B tracer has an inert-like transport behavior in salt pans and does not easily form complexes or precipitates at the brine–mineral interface. The tracer began to break through after a cumulative injection of 1.3 PV and was nearly completely expelled after 6.5 PV, indicating minimal adsorption in the simulation. The curve dynamics are related to the particle size and surface layer of the packing material. In fine salt layers, the uniform pore sizes and straight paths allow the tracer to move quickly with the displacement fluid, creating a smooth, complete outflow curve that shows “uniform spreading with the displacement fluid.”^31,32^ In kosher salt layers, the non-connected pores from large salt particles cause the tracer to flow intermittently in local channels, leading to irregular step-like fluctuations in the outflow curve.^33^ In silt layers, the small, winding pores increase the tracer's travel difficulty, trapping much of it in complex channels. The resulting breakthrough curve has the typical feature of a contracted peak.

This study further reveals through flow experiment data that the loss rates of the ^10^B tracer in fine salt, coarse salt, and mud are 11.7% ± 1.2%, 13.4% ± 1.5%, and 16.8% ± 2.1%, respectively, as shown in Fig. 7(d). The gradual increase in the ^10^B tracer's loss rate across different media is fundamentally attributed to differences in specific surface area.^34,35^ The fine salt medium, composed of millimeter-sized crystal particles, has a smooth surface and homogeneous pores, resulting in the smallest specific surface area per unit mass. The coarse salt medium, with micrometer-scale roughness and secondary fractures on its particle surfaces, has a specific surface area 30–50% larger than that of fine salt, providing more interfaces for physical adsorption. The mud medium, due to its honeycomb-like pore network of clay minerals, has a specific surface area several times larger than that of coarse salt. This increase in specific surface area enhances the collision frequency and contact time between solute molecules and the medium surface, resulting in a stepwise increase in the loss rate from fine salt to mud. These results provide critical design parameters for practical applications. Under typical brine seepage conditions in salt pans, the tracer dosage needs to be increased by 20% to compensate for adsorption losses by the medium.

**Fig. 8:**
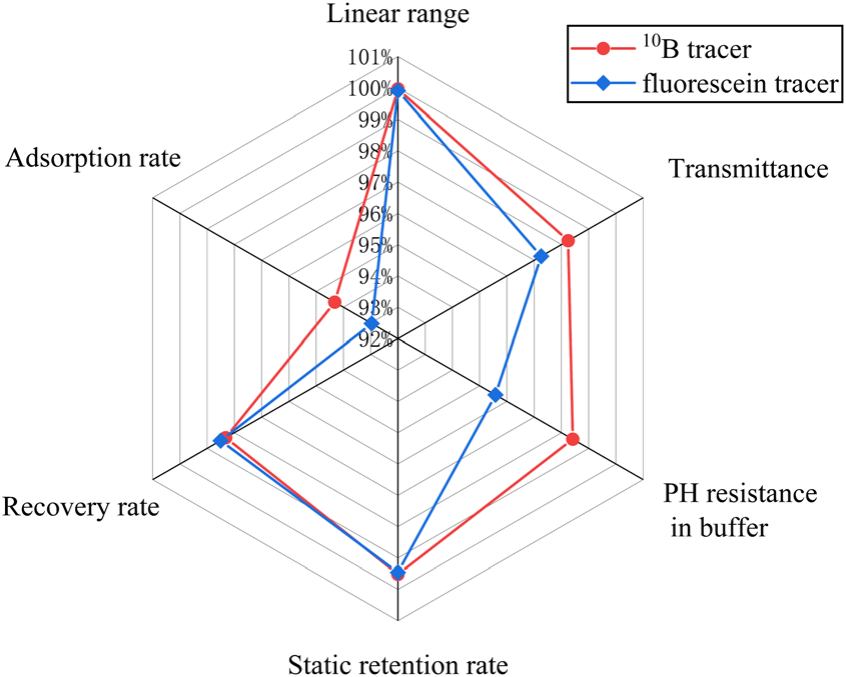
Performance comparison of ^10^B tracer and fluorescein tracer.

As shown in Fig. 8, both the ^10^B tracer and fluorescein exhibit good performance. However, the ^10^B tracer outperforms fluorescein, especially in adsorption rate and buffer pH resistance, demonstrating more advantages in these metrics.

### 3.3 Spatial heterogeneity of seepage systems in field experiments

**Fig. 9:**
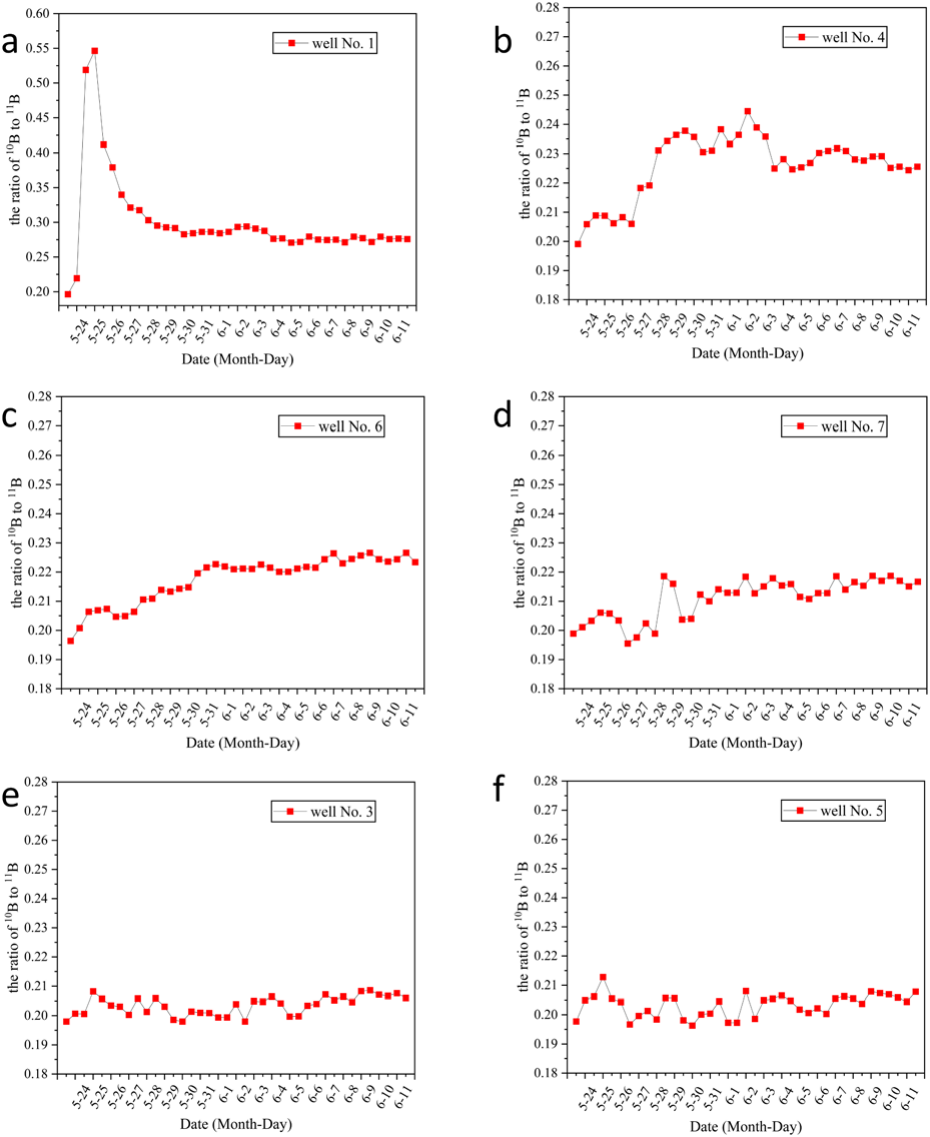
Temporal variation of ^10^B/^11^B ratio in monitoring wells: (a) well 1; (b) well 4; (c) well 6; (d) well 7; (e) well 3; (f) well 5.

Analysis of inductively coupled plasma mass spectrometry data revealed distinct tracer response characteristics across monitoring wells. As shown in Fig. 9(a)–(c), well 1 exhibited a pronounced tracer response: the ^10^B/^11^B ratio rapidly surged from a background value of 0.197 to a peak of 0.546 on the day following injection, stabilizing thereafter at 0.275. This indicates well 1 as a preferential seepage channel, with tracer breakthrough completed within 24 hours. In contrast, well 3, positioned 4 m from the injection point, exhibited only minor isotopic ratio fluctuations, stabilizing from an initial 0.198 to 0.208, indicating it is not a primary seepage pathway. Monitoring well no. 4 demonstrates a unique dynamic response pattern. The isotopic ratio peaked at 0.246 on day 10, followed by oscillatory attenuation before stabilizing at 0.224. This behavior suggests coexisting inflow and outflow dynamics within the well, though fluid fluxes remain challenging to quantify. The observed periodic imbalance between tracer influx and efflux fluxes, likely attributable to fracture networks in salt layers and localized pore clogging, drives the nonlinear decay pattern.^36–38^ These findings provide critical insights into fluid migration behaviors in complex geological environments.

The data analysis from Fig. 9(d)–(f) reveals that the ^10^B/^11^B ratio in well 5 experienced a brief peak of 0.215 before stabilizing at 0.208. This delayed response and the absence of a sustained upward trend suggest that well 5 is located at the edge of the seepage system. In contrast, well 7 exhibited a slight peak of 0.220 on the sixth day and eventually stabilized at 0.218. This limited fluctuation indicates the presence of a low-permeability geological barrier in its seepage pathway, restricting fluid flow.^39,40^ Notably, the ^10^B/^11^B ratio in well 6 showed a continuous upward trend without stabilizing, suggesting significant seepage retention in the area. This irregular variation implies that fluid flow may be slowed or halted in certain geological structures, leading to long-term changes in the isotopic ratio. It can therefore be inferred that well 6 is likely a leaking well, and appropriate anti-seepage measures should be implemented to prevent further leakage.

**Fig. 10:**
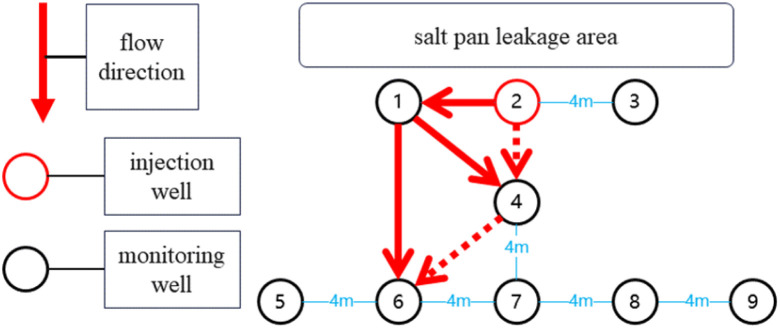
Field-scale mobility simulation results.

Based on the data analysis and the illustration shown in Fig. 10, the main seepage path of the salt field leakage system is characterized by fluid primarily flowing rapidly along the pathway from 2 → 1 → 6. This phenomenon indicates that the fracture system plays a dominant role in fluid migration. The isotopic ratio at well 1 rose rapidly the day after injection and remained at a high level, suggesting the existence of a dominant fracture channel. The formation of this channel is attributed to the combined effects of salt layer dissolution and geological structure, exhibiting characteristics of low resistance and high velocity in seepage.^41,42^ The stable isotope ratio at well 6 continued to rise steadily. Substituting the parameters *s* = 1.2 m, *Q* = 2.832 m^3^ d^−1^, *t* = 1.04 × 10^−3^ d, and *r* = 8.94 m into formula (3) yielded a hydraulic conductivity *k* of 0.0978 m d^−1^. This suggests the potential presence of a weakly enclosed area with medium to high permeability downstream. The rapid response and stable flow characteristics of the main seepage path further confirm the high connectivity of the fracture system. In contrast, at well no. 3, a monitoring well located 4 meters from the injection point, the signal strength was barely detectable. Therefore, this paper calculated the Peclet number for this well. Based on literature and measured data, the relevant parameters are as follows: diffusion coefficient *D*_0_ for ^10^B molecules at 0.45 nm = 5.45 × 10^−10^ m^2^ s^−1^, Darcy flow velocity *v* = 4 × 10^−9^ m s^−1^, tortuosity *τ* = 2.1, and porosity *ϕ* = 0.2. The Peclet number was calculated using formula (2), yielding Pe >1. These results confirm that solute transport is dominated by advection, overriding the effects of molecular diffusion. Therefore, this paper suggests that the absence of tracer ^10^B inflow in well no. 3 is primarily attributed to the action of the assisted plugging mechanism. This finding, verified by inversion analysis, further confirms the significant hydraulic advantage of the main seepage channel at well 1. The formation of the auxiliary path 2 → 1 → 4 → 6 stems from the synergistic effect of fractures and tiny pores. The fluid concentration at well 4 first fluctuates and then stabilizes, indicating that while the main fractures rapidly transport fluids, the surrounding tiny fractures and low-permeability pores temporarily “store and release” fluids, causing concentration fluctuations. This path is essentially a branch of the main fracture network extending from well 1, where fluid transport is accomplished through the cooperation of tiny fractures and pores, yet its transport efficiency is lower than that of the main channel. The differences between the main and auxiliary paths are mainly influenced by three factors: the orientation of fracture distribution, the uneven distribution of pores, and the dynamic changes of blockages.^43,44^ These findings provide vital references for precisely locating seepage plugging projects. Simple anti-seepage measures were implemented at well 6 in this study. Measurement and calculation results revealed a decrease in the leakage coefficient from 0.0684 m d^−1^ to 0.0447 m d^−1^, confirming the presence of leakage at this well. To achieve further leakage reduction, more efficient anti-seepage equipment would be required.

## 4. Conclusions

This study addresses the technical challenges in brine seepage monitoring by pioneering the application of the ^10^B isotope tracer technology, establishing a high-precision, multi-dimensional dynamic monitoring system. This system provides a scientific foundation for the accurate identification of seepage pathways, analysis of flow field heterogeneity, and engineering anti-seepage design. Through systematic laboratory validation, the ^10^B tracer demonstrated multi-dimensional performance advantages in ultra-high salinity brine systems: detection sensitivity at the 10^−9^ level enables effective capture of trace-level leakage signals; stable recovery rates ranging from 92.8% to 106.5% comply with international standards for trace element analysis; adsorption loss below 6.31% significantly outperforms conventional tracers; and light transmittance and acid-base resistance retention rates exceeding 95% confirm its long-term stability under extreme salinity and complex chemical conditions. Compared to the fluorescein control test, the ^10^B tracer demonstrated greater advantages in adsorption rate and buffered pH resistance. Mobility experiments further revealed the inert-like transport characteristics of the ^10^B tracer, with breakthrough curves highly consistent with those of typical inert ions and retention loss below 20%, demonstrating its capability to precisely characterize fluid migration in heterogeneous porous media. Adsorption kinetic analyses across fine salt, coarse salt, and silt media identified multi-scale physicochemical mechanisms underpinning the tracer's anti-adsorption performance, offering theoretical support for dynamic optimization of tracer dosage in engineering practice.

Field experiments successfully uncovered the spatial heterogeneity and dynamic evolution of the salt pan seepage system. The main seepage pathway 2 → 1 → 6, dominated by a highly connected fracture network, exhibited rapid breakthrough, stable flow velocity, and sustained concentration response, highlighting the hydraulic control advantages of the fracture system. The secondary pathway 2 → 1 → 4 → 6, regulated by the coupling of fractures and micropores, displayed oscillatory concentration patterns, low transmission efficiency, and periodic retention, reflecting the critical influence of geological heterogeneity on seepage pathway differentiation. For well 3, Peclet number calculation yielded Pe >1, with absent ^10^B tracer inflow primarily caused by an auxiliary plugging mechanism. Delayed responses and weak signals in peripheral monitoring wells such as well 5 and well 7 further confirmed the presence of permeability barriers, likely formed by dense clay layers or localized salt cementation, providing direct evidence for targeted anti-seepage engineering. Following the implementation of simple anti-seepage measures at well 6, the leakage coefficient decreased, confirming the presence of leakage at this well. More effective anti-seepage equipment is required to further reduce seepage. This technology overcomes the limitations of traditional methods in detection accuracy, real-time capability, and quantitative analysis. By integrating high-resolution tracer data with dynamic flow field, it achieves a technological leap from empirical estimation to precise diagnosis, offering an innovative tool for efficient salt lake resource exploitation, leakage risk mitigation, and ecological anti-seepage design.

Future research could focus on three directions: first, integrating artificial intelligence algorithms and IoT technologies to develop a real-time seepage monitoring and intelligent early-warning platform for dynamic leakage event detection and rapid response. Second, conducting multi-field coupled simulations to reveal the cumulative impacts of long-term mining activities on salt layer permeability. Third, expanding the application of the ^10^B tracer technology to other high-salinity environments to validate its universality and engineering potential. Through technological iteration and interdisciplinary collaboration, this method is poised to become a core technology for salt lake ecosystem conservation and sustainable resource utilization, supporting the efficient development of strategic salt lake resources and ecological security under the dual-carbon goals.

## Author contributions

Conceptualization, Q. W.; methodology, M. S.; validation, L. W.; formal analysis, Y. D.; investigation, Z, L.; resources, L. Z. and L. L.; data curation, H, W.; writing original draft, J. H.; writing – review and editing, Q. W.; supervision, Q. W.; P. All authors have read and agreed to the published version of the manuscript.

## Conflicts of interest

The authors declare that they have no known competing financial interests or personal relationships that could have appeared to influence the work reported in this paper.

## Data Availability

Data for this article is available at Science Data Bank at https://doi.org/10.57760/sciencedb.23897.
